# 2,4-Dichloro-7-fluoro­quinazoline

**DOI:** 10.1107/S1600536812006125

**Published:** 2012-02-17

**Authors:** Feng Gao, Yun-Fei Hu, Jin-Long Wang

**Affiliations:** a Department of Chinese Traditional Herbs, Agromomy College, Sichuan Agricultural University, Chengdu 611130, People’s Republic of China

## Abstract

The mol­ecule of the title compound, C_8_H_3_Cl_2_FN_2_, is essentially planar, with a maximum deviation of 0.018 (2) Å. In the crystal, π–π stacking is observed between parallel quinazoline moieties of adjacent mol­ecules, the centroid–centroid distance being 3.8476 (14) Å.

## Related literature

For the synthesis of quinazoline derivatives, see: Roberts *et al.* (2011 ▶); Gao *et al.* (2010 ▶); Li *et al.* (2009 ▶); Connolly *et al.* (2005 ▶). For the pharmacological properties of quinazoline analogues, see: Koller *et al.* (2011 ▶); Garofalo *et al.* (2011 ▶); Yang *et al.* (2011 ▶). For related structures, see: Jia *et al.* (2011 ▶); Ouahrouch *et al.* (2011 ▶).
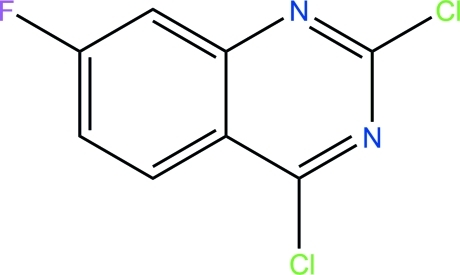


## Experimental

### 

#### Crystal data

C_8_H_3_Cl_2_FN_2_
*M* *_r_* = 217.02
Monoclinic, 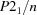

*a* = 3.8257 (3) Å
*b* = 15.0664 (9) Å
*c* = 14.3453 (6) Å
β = 95.102 (5)°
*V* = 823.59 (9) Å^3^
*Z* = 4
Mo *K*α radiation
μ = 0.75 mm^−1^
*T* = 293 K
0.35 × 0.30 × 0.25 mm

#### Data collection

Agilent Xcalibur Eos diffractometer
Absorption correction: multi-scan (*CrysAlis PRO*; Agilent, 2010 ▶) *T*_min_ = 0.780, *T*_max_ = 0.835
3156 measured reflections
1452 independent reflections
1120 reflections with *I* > 2σ(*I*)
*R*_int_ = 0.025

#### Refinement

*R*[*F* ^2^ > 2σ(*F* ^2^)] = 0.038
*wR*(*F* ^2^) = 0.095
*S* = 1.07
1452 reflections
118 parameters
H-atom parameters constrained
Δρ_max_ = 0.20 e Å^−3^
Δρ_min_ = −0.23 e Å^−3^

### 

Data collection: *CrysAlis PRO* (Agilent, 2010 ▶); cell refinement: *CrysAlis PRO*; data reduction: *CrysAlis PRO*; program(s) used to solve structure: *SHELXTL* (Sheldrick, 2008 ▶); program(s) used to refine structure: *SHELXTL*; molecular graphics: *SHELXTL*; software used to prepare material for publication: *SHELXTL*.

## Supplementary Material

Crystal structure: contains datablock(s) I, global. DOI: 10.1107/S1600536812006125/xu5446sup1.cif

Structure factors: contains datablock(s) I. DOI: 10.1107/S1600536812006125/xu5446Isup2.hkl

Supplementary material file. DOI: 10.1107/S1600536812006125/xu5446Isup3.cml

Additional supplementary materials:  crystallographic information; 3D view; checkCIF report

## Figures and Tables

**Fig. 1.**
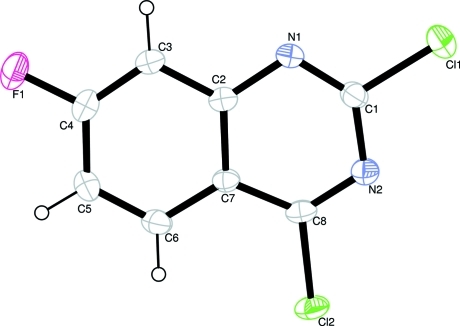
Molecular structure of showing 30% probability displacement ellipsoids.

**Fig. 2.**
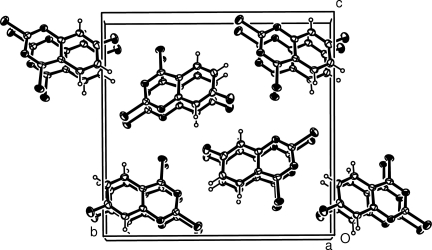
The packing viewed along c axis with π···π interactions, indicating the dimer.

## supplementary crystallographic information

### Comment

As the important six-membered heterocycles, quinazoline derivatives have always drawn the attention of organic and medicinal chemists for their various biological activities and significant synthetic materials. Several quinazoline-containing compounds have been approved by FDA, such as EGFR inhibitors Iressa and Tarceva used in the treatment of cancer, as well as alpha adrenergic receptor antagonists Praosin and Alfuzosine, which have been used for anti-hypertensive drugs in clinic. In addition, quinazoline derivatives also have been reported as anti-inflammatory, antibacterial or anticonvulsant agents. Most recently, quinazoline analogues have been found as selective VEGFR-2 receptor tyrosine kinase inhibitors, novel heat shock protein 90 inhibitors, EGFR Tyrosine kinase inhibitors, as well as glucocerebrosidase inhibitors.

### Experimental

2,4-Dichloro-7-fluoroquinazoline was synthesized presently through two steps reactions implying 2-amino-4-fluorobenzoic acid as the starting material: 1. Acetic acid (80 ml) was added to a suspension of 2-amino-4-fluorobenzoic acid (100 g, 0.645 mol) in water (2*L*), a solution of NaOCN (105 g, 1.616 mol) in water (800 ml) was added dropwise under vigorous stirring with a mechanical stirrer. The reaction mixture was stirred at room temperature for 30 min, and NaOH (480 g, 12 mol) was added in small portions, and the mixture was cooled to room temperature. Then concentrated HCl (~1.2*L*) was added dropwise to the reaction mixture to attain pH ~4 (strong foaming!). The formed precipitate was separated by filtration, washed with water, and air-dried to give compound 7-fluoroquinazoline-2,4(1*H*,3*H*)-dione (yield 82%, 95 g). It was used in the next step without any purification. 2. A mixture of compound 7-fluoroquinazoline-2,4(1*H*,3*H*)-dione (150 g, 0.83 mol), *N*,*N*-diethylaniline (125 g, 0.84 mol), and POCl_3_ (500 ml) was refluxed for overnight. Most of POCl_3_ was removed by rotary vapor. The residue was poured into the mixture water/ice (~4*L*), and the formed precipitate was separated by filtration, washed with water, and vacuum-dried to give compound 2,4-dichloro-7-fluoroquinazoline (yield 94%, 170 g). Single crystals of 2,4-dichloro-7-fluoroquinazoline, C_8_H_3_Cl_2_FN_2_, were recrystallized from acetone, mounted in inert oil and transferred to the cold gas stream of the diffractometer.

### Refinement

H atoms were placed in calculated positions and treated as riding atoms [C—H = 0.93–0.96 Å], with *U*_iso_(H) = 1.5*U*_eq_(C) for methyl H atoms and 1.2*U*_eq_(C) for others.

### Crystal data

**Table:**

C_8_H_3_Cl_2_FN_2_	*F*(000) = 432
*M**_r_* = 217.02	*D*_x_ = 1.750 Mg m^−^^3^
Monoclinic, *P*2_1_/*n*	Mo *K*α radiation, λ = 0.71073 Å
*a* = 3.8257 (3) Å	Cell parameters from 1033 reflections
*b* = 15.0664 (9) Å	θ = 3.0–25.0°
*c* = 14.3453 (6) Å	µ = 0.75 mm^−^^1^
β = 95.102 (5)°	*T* = 293 K
*V* = 823.59 (9) Å^3^	Block, brown
*Z* = 4	0.35 × 0.30 × 0.25 mm

### Data collection

**Table:**

Agilent Xcalibur Eos diffractometer	1452 independent reflections
Radiation source: Enhance (Mo) X-ray Source	1120 reflections with *I* > 2σ(*I*)
Graphite monochromator	*R*_int_ = 0.025
Detector resolution: 10.0 pixels mm^-1^	θ_max_ = 25.0°, θ_min_ = 3.1°
ω scans	*h* = −4→4
Absorption correction: multi-scan (*CrysAlis PRO*; Agilent, 2010)	*k* = −15→17
*T*_min_ = 0.780, *T*_max_ = 0.835	*l* = −16→16
3156 measured reflections	

### Refinement

**Table:**

Refinement on *F*^2^	Primary atom site location: structure-invariant direct methods
Least-squares matrix: full	Secondary atom site location: difference Fourier map
*R*[*F*^2^ > 2σ(*F*^2^)] = 0.038	Hydrogen site location: inferred from neighbouring sites
*wR*(*F*^2^) = 0.095	H-atom parameters constrained
*S* = 1.07	*w* = 1/[σ^2^(*F*_o_^2^) + (0.0389*P*)^2^] where *P* = (*F*_o_^2^ + 2*F*_c_^2^)/3
1452 reflections	(Δ/σ)_max_ < 0.001
118 parameters	Δρ_max_ = 0.20 e Å^−^^3^
0 restraints	Δρ_min_ = −0.23 e Å^−^^3^

### Special details

**Table:**

Geometry. All e.s.d.'s (except the e.s.d. in the dihedral angle between two l.s. planes) are estimated using the full covariance matrix. The cell e.s.d.'s are taken into account individually in the estimation of e.s.d.'s in distances, angles and torsion angles; correlations between e.s.d.'s in cell parameters are only used when they are defined by crystal symmetry. An approximate (isotropic) treatment of cell e.s.d.'s is used for estimating e.s.d.'s involving l.s. planes.
Refinement. Refinement of *F*^2^ against ALL reflections. The weighted *R*-factor *wR* and goodness of fit *S* are based on *F*^2^, conventional *R*-factors *R* are based on *F*, with *F* set to zero for negative *F*^2^. The threshold expression of *F*^2^ > σ(*F*^2^) is used only for calculating *R*-factors(gt) *etc*. and is not relevant to the choice of reflections for refinement. *R*-factors based on *F*^2^ are statistically about twice as large as those based on *F*, and *R*- factors based on ALL data will be even larger.

### Fractional atomic coordinates and isotropic or equivalent isotropic displacement parameters (Å^2^)

**Table:**

	*x*	*y*	*z*	*U*_iso_*/*U*_eq_	
Cl1	0.18763 (19)	0.24582 (4)	0.86579 (4)	0.0500 (3)	
Cl2	0.2734 (2)	0.09054 (5)	0.54819 (5)	0.0628 (3)	
F1	0.8584 (4)	0.55046 (10)	0.60324 (11)	0.0652 (5)	
N1	0.2491 (5)	0.17987 (14)	0.70176 (14)	0.0416 (5)	
N2	0.4812 (5)	0.25290 (13)	0.57255 (14)	0.0389 (5)	
C1	0.3448 (6)	0.18701 (17)	0.61375 (17)	0.0392 (6)	
C2	0.3050 (6)	0.25134 (16)	0.75263 (17)	0.0360 (6)	
C3	0.4494 (6)	0.33043 (16)	0.72000 (16)	0.0331 (6)	
C4	0.5323 (6)	0.32793 (16)	0.62626 (16)	0.0327 (6)	
C5	0.6758 (7)	0.40354 (16)	0.58670 (16)	0.0389 (6)	
H5	0.7365	0.4030	0.5253	0.047*	
C6	0.7228 (7)	0.47677 (17)	0.64004 (19)	0.0428 (7)	
C7	0.6403 (7)	0.48233 (17)	0.73259 (19)	0.0455 (7)	
H7	0.6783	0.5346	0.7665	0.055*	
C8	0.5030 (7)	0.40973 (16)	0.77230 (17)	0.0426 (7)	
H8	0.4443	0.4122	0.8338	0.051*	

### Atomic displacement parameters (Å^2^)

**Table:**

	*U*^11^	*U*^22^	*U*^33^	*U*^12^	*U*^13^	*U*^23^
Cl1	0.0595 (5)	0.0612 (5)	0.0313 (4)	0.0047 (4)	0.0158 (3)	0.0092 (3)
Cl2	0.0870 (6)	0.0476 (5)	0.0541 (5)	−0.0111 (4)	0.0086 (4)	−0.0150 (4)
F1	0.0783 (13)	0.0466 (10)	0.0717 (12)	−0.0159 (9)	0.0123 (10)	0.0132 (9)
N1	0.0454 (14)	0.0428 (13)	0.0367 (12)	−0.0035 (11)	0.0043 (10)	0.0014 (11)
N2	0.0455 (13)	0.0404 (13)	0.0314 (11)	−0.0004 (11)	0.0074 (10)	−0.0032 (10)
C1	0.0433 (16)	0.0373 (15)	0.0369 (14)	0.0015 (13)	0.0029 (12)	−0.0033 (12)
C2	0.0330 (14)	0.0469 (16)	0.0285 (12)	0.0071 (12)	0.0045 (11)	0.0052 (12)
C3	0.0320 (14)	0.0385 (14)	0.0286 (12)	0.0057 (12)	0.0022 (11)	0.0039 (11)
C4	0.0301 (14)	0.0365 (14)	0.0315 (13)	0.0033 (12)	0.0019 (11)	0.0037 (11)
C5	0.0427 (16)	0.0437 (16)	0.0311 (13)	0.0022 (13)	0.0074 (12)	0.0037 (12)
C6	0.0389 (15)	0.0397 (16)	0.0493 (16)	−0.0033 (13)	0.0008 (13)	0.0123 (14)
C7	0.0504 (17)	0.0370 (15)	0.0485 (16)	0.0004 (13)	0.0013 (13)	−0.0069 (13)
C8	0.0469 (17)	0.0478 (16)	0.0336 (14)	0.0060 (14)	0.0066 (12)	−0.0037 (13)

### Geometric parameters (Å, º)

**Table:**

Cl1—C2	1.724 (2)	C3—C8	1.416 (3)
Cl2—C1	1.739 (3)	C4—C5	1.406 (3)
F1—C6	1.352 (3)	C5—C6	1.346 (3)
N1—C2	1.308 (3)	C5—H5	0.9300
N1—C1	1.350 (3)	C6—C7	1.394 (4)
N2—C1	1.289 (3)	C7—C8	1.360 (3)
N2—C4	1.372 (3)	C7—H7	0.9300
C2—C3	1.411 (3)	C8—H8	0.9300
C3—C4	1.409 (3)		
			
C2—N1—C1	114.3 (2)	C5—C4—C3	119.5 (2)
C1—N2—C4	114.9 (2)	C6—C5—C4	118.2 (2)
N2—C1—N1	130.1 (2)	C6—C5—H5	120.9
N2—C1—Cl2	116.50 (18)	C4—C5—H5	120.9
N1—C1—Cl2	113.36 (19)	C5—C6—F1	119.2 (2)
N1—C2—C3	124.0 (2)	C5—C6—C7	124.1 (2)
N1—C2—Cl1	116.25 (18)	F1—C6—C7	116.7 (2)
C3—C2—Cl1	119.70 (18)	C8—C7—C6	118.6 (2)
C4—C3—C2	115.0 (2)	C8—C7—H7	120.7
C4—C3—C8	119.6 (2)	C6—C7—H7	120.7
C2—C3—C8	125.4 (2)	C7—C8—C3	120.0 (2)
N2—C4—C5	118.8 (2)	C7—C8—H8	120.0
N2—C4—C3	121.7 (2)	C3—C8—H8	120.0
